# Sustainable Supply Chain Management in the Food Industry: A Conceptual Model from a Literature Review and a Case Study

**DOI:** 10.3390/foods11152295

**Published:** 2022-08-01

**Authors:** Theofilos Mastos, Katerina Gotzamani

**Affiliations:** Department of Business Administration, University of Macedonia, 54636 Thessaloniki, Greece; kgotza@uom.edu.gr

**Keywords:** sustainable supply chain management, food industry, literature review, case study, Greece

## Abstract

The purpose of this study is twofold: firstly, to provide a literature review of sustainable supply chain management (SSCM) critical factors, practices and performance; and secondly, to develop a comprehensive and testable model of SSCM in the food industry. The research conducted comprises a literature review and a case study. The literature review findings propose a theoretical framework linking SSCM critical factors, practices and performance. The case study comprises two sustainability leaders in the Greek food supply chain in order to investigate the three SSCM constructs. A new set of pioneering SSCM practices in the Greek food industry is identified, including daily conversation, local sourcing and HR investments. The end result of this research proposes a testable model that sheds light on SSCM in the food industry and is based on a set of propositions.

## 1. Introduction

Over the past decades, sustainable supply chain management (SSCM) has attracted much attention from academics and practitioners [1,2]. Globalisation allowed processes to be dispersed around the world, linking all supply chain members, from suppliers to end customers, through information sharing and material and capital flows [2]. As a result, pressures from stakeholders, such as regulatory bodies, non-governmental organisations (NGOs), community organizations, suppliers, customers and global competition, have prompted companies to reconsider the balance of environmental, social and economic issues in their supply chains [3] and adopt sustainable supply chain management practices. SSCM is defined as “the management of material, information and capital flows as well as cooperation among companies along the supply chain while taking goals from all three dimensions of sustainable development, i.e., economic, environmental and social, into account which are derived from customer and stakeholder requirements” [2] (p. 1700).

As in all business operations, SSCM tries to achieve clearly defined performance goals [4]. However, this is not an easy task due to the complexity of supply chains, where individual members have different and often conflicting goals from other members of the chain and hence different performance measures. Different measures are not always seen as positive regarding the entire chain’s performance, because a single company’s outcomes may be harmful for other supply chain members. Hence, the performance of the entire chain can only be improved if the supply chain is conceptualized as a whole, outside the boundaries of the firm level [5].

SSCM practices such as environmental purchasing and sustainable packaging often have positive outcomes regarding supply chain sustainability performance [6]. The development of SSCM practices can either be enabled or inhibited by various contingent factors. A variety of industries face specific enabling or inhibiting factors from different points of view based on their size, culture, location and supply chain partners. Many researchers have studied SSCM in several sectors such as manufacturing [2,7], the automotive industry [8], oil and gas [9], energy [10] and the food industry [11]. The food industry is one of the most important sectors that faces significant environmental, economic, social and political challenges. This is due to the focus of public attention on food safety, production practices, environmental issues such as deforestation, climate change and energy consumption and social issues such as fair wages and population growth [11,12]. Furthermore, globalization, technological advances, the use of agricultural chemicals and improved transportation have simultaneously raised concerns regarding the sustainability of food supply chains [13,14], since “changes at one stage in a supply chain will have knock-on effects on other stages in the chain” [14] (p. 97).

Other critical issues are related to the measurement of supply chain impacts, to supply chain collaboration and networking, to stakeholder engagement, to sustainable development goals, etc. [15]. These challenges confirm the differentiability of food supply chains, which lies upon variability and risk factors due to the product-specific characteristics such as perishability, seasonality in production, transportation and storage conditions [16]. In addition, customers and firms have raised their concerns regarding the origin of products, food safety, quality and sustainable production [17], including animal welfare and environmental pressure [16].

Numerous studies have investigated the relationship between SSCM practices and sustainability performance. However, limited work has been conducted on the empirical investigation of industry and location-specific SSCM critical factors and practices and their relationship to sustainability performance [11,18,19,20,21]. The food industry is characterised by enhanced supply chain relationships that aim at achieving high sustainability performance [11]. Earlier findings from ref. [6] demonstrate that environmentally friendly purchasing and sustainable packaging result in improved economic and social performance. Direct and indirect impacts between the dimensions of sustainability performance are also observed in the literature. A positive relationship is found between corporate social performance and financial performance [22]. In the wine industry, ref. [23] found that employee practices related to social sustainability result in reduced costs; ref. [24] found that environmental practices have positive environmental performance outcomes and indirect impacts on cost performance based on quality improvements. The authors of ref. [25] suggest an alignment between goals that lead to improved environmental and financial performance. On the other hand, ref. [24] highlights “the complexity of sustainability impacts on performance and suggest that performance benefits from sustainability programs may be difficult to recognize” [24] (p. 38).

With the above in mind, the aim of this study is to gain insight into the SSCM critical factors and practices that are implemented in the food industry and their possible relationship to sustainability performance. To support the purpose of this research, two methods were used. A literature review of the key SSCM topics and a case study to demonstrate the experience of two leaders in SSCM. The aim of this research will be achieved by addressing the following research questions (RQ):

RQ1: What are the factors that influence the adoption of SSCM practices in the food industry?

RQ2: Which practices do companies in the food industry adopt to develop SSCM?

RQ3: What measures can be used to measure SSCM performance in the food industry?

The rest of the paper is organized as follows. The next section presents the literature review and the case study methods. The results of the literature review and the case study are presented and discussed in conjunction with previous research in Section 3. Finally, the conclusions are drawn in Section 4, including the study limitations as well as future research opportunities.

## 2. Materials and Methods

The research methodology that was applied in this study is based on the following steps [15]: (i) a literature review; (ii) identification of the gaps; (iii) concepts synthesis; and (iv) a case study.

### 2.1. Literature Review Method

Because the identification and conceptualisation of SSCM is still unclear, a literature review was conducted on the key sustainable supply chain management topics, such as critical factors for implementation, practices and performance. Despite the fact that other reviews on the SSCM are already published, this review is required in order provide an up-to-date report and understanding of the current SSCM research. The search for related scientific articles was based on keywords and authors’ names, in major bibliographical databases and publishers such as Scopus, Elsevier, Emerald, Springer, Wiley, Taylor & Francis, Springer, Sage Publications and Inderscience, over a twenty-year period since 2000. The keywords search included “sustainable supply chain management”, “drivers”, “barriers”, “enablers”, “motivators”, “critical factors”, “sustainable supply chain management practices”, “sustainability performance” and “food industry”. The authors search included Seuring S., Beske P., Gualandirs J., Govindan, K, Pagell M., etc., since these authors have repeatedly focused their research on SSCM topics [1]. A secondary search was also carried out using the cited references. Only papers in peer-reviewed English scientific journals are reviewed. This research includes articles with a focus on the food industry as a field of application but is not limited to that. Articles from other sectors were also included in the study.

The measures identified by the comprehensive literature review were named and grouped based on the affinity method, which is utilized to organize into categories common themes from a large amount of information [26]. In addition to the affinity method, the naming and grouping of the constructs were based on interviews of five professionals of the food industry and five academics.

### 2.2. Case Study Method

Taking into account that the analysis of a supply chain as a whole is a complex and difficult task and in order to explore the SSCM critical factors, practices and performance in the food industry, a case study was selected as the most appropriate research method [27,28]. This study investigates a sustainable supply chain in order to capture the critical factors of SSCM, the SSCM practices adopted and their influence on sustainability performance. The research has been carried out in a supply chain that is comprised of two SSCM leaders that operate in Greece (Table 1). This is particularly useful, because it offers empirical contributions within the Greek-business context, where SSCM literature is limited. The names of the companies were not disclosed in order to protect confidentiality and encourage the openness of responses. The unit of analysis in this study is the food supply chain. The study investigates the particular food supply chain, comprised of two companies, and the findings will concern the supply chain as a whole.

**Figure 1:**
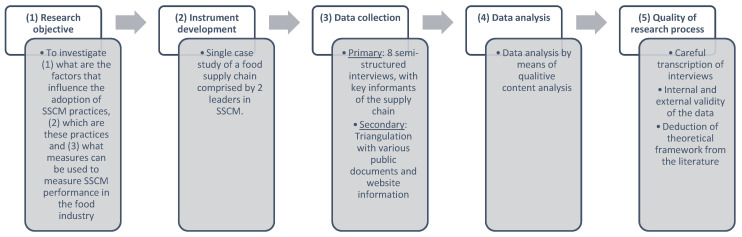
The five stages of the research process model.

The authors of ref. [29] propose a five-stage process for case studies that is used for structuring this research. Figure 1 depicts the various research steps.

1. The first step is related to the research objective. This research uses a single case study to investigate the critical factors that influence companies in the food industry to implement SSCM practices, which are these practices and how do they influence sustainability performance.
2. The second step is related to the research instrument development. A single case research design is used to guide this study and provide an in-depth understanding of a complex phenomenon, through the observation of actual practices in real-world settings, without any kind of control or manipulation, considering both temporal and contextual dimensions [30,31]. Case studies provide researchers the opportunity to closely analyse the data within a specific context. In ref. [32] (p. 18), the authors define the case study research method “as an empirical inquiry that investigates a contemporary phenomenon within its real-life context; when the boundaries between phenomenon and context are not clearly evident; and in which multiple sources of evidence are used.” Furthermore, the detailed qualitative accounts often produced in case studies not only help to explore or describe the data in real-life environments but also help to explain the complexities of real-life situations, which may not be captured through experimental or survey research [33]. For the reasons referred to above, a single case study comprised by two leaders in the food industry was selected as the most appropriate research method for this study. The firms are both sustainability leaders in the Greek food industry and members of multinational groups. The companies were selected as they have received a series of recognitions regarding sustainability, such as Environmental Awards, Supply Chain Sustainability Awards, distinctions in CSR actions, etc. Furthermore, both companies play a crucial role in the Greek industry, society and economy. An interview protocol [27] was developed on the basis of the reviewed literature and closely following previous research on SSCM [34,35] (see Appendix A). The authors of ref. [36] highlight that using existing questions enables the comparability of results. Furthermore, ref. [28] points out that using interview protocols assures the reliability of data. The interviews ranged from 70 to 90 min.

3. The third step includes data collection. The sources of information included 8 face-to-face interviews with
  (1) The CSR Manager of the SB company;
  (2) The Quality Manager of the SB company;
  (3) The Manager of the distribution centre of the SM company;
  (4) Two Logistics Project Managers of the SM company;
  (5) Three Retail Store Managers of the SM company.

Field notes were typed up during each interview. Repeated contacts by phone or e-mails were needed to confirm the chain of evidence. Except for the data drawn from interviews, the analysis of the sustainability reports in combination with the website information and other news were important secondary sources.

4. The fourth step refers to the data analysis. The data analysis was filtered and guided by the identified SSCM constructs.

The fifth step is related to assuring the quality of the research process: Multiple sources of data were collected, including archival data (financial reports, CSR reports, website material and company records), on-site observations and semi-structured interviews, in order to achieve data source triangulation and ensure construct validity ([37], p. 68; [28], p. 36). The internal validity of the case was assured by doing pattern matching with other studies identified in previous research [28]. Regarding the external validity, the case study was designed and conducted based on the gathering of as many data as possible in order to attain a deeper knowledge of the complex background of SSCM and to identify the more analytical and general theoretical implications [28].

## 3. Results and Discussion

This section begins with the literature review results, highlighting the concepts of the sustainable supply chain management and continues with the case study results of the food supply chain.

### 3.1. Literature Review Results

The results of the literature review are classified in three main SSCM content categories, namely, critical factors, practices and performance.

#### 3.1.1. Critical Factors

In studying the literature, many terms are found to be used interchangeably by researchers. For example, the factor top management commitment is considered as enabler [35,38], driver [39], success factor [40], critical factor [41], enabling factor [42], reason [43], motivator [19] and firm-level strength [44]. In contrast, most researchers in the SSCM literature use the term barrier when describing factors that inhibit SSCM, such as the lack of top management commitment [1,35,39]. As observed, there are several terms to describe the same factor, indicating a lack of agreement on how these terms should be used in SSCM research. Furthermore, these factors are classified in more than one category, such as internal and external [35,45,46], regulatory, resource, market and social [47], stakeholder, process or product [48].

The identified factors are named critical factors, including enablers, drivers, success factors, motives as well as barriers and inhibiting factors. More specifically, in this study critical factors are defined as the factors that are responsible for enabling or inhibiting the successful implementation of SSCM. This is the rationale for grouping the enablers, drivers, success factors, motives, barriers and inhibiting factors in one group. This approach is also applied in other studies that investigate SSCM [41]. A total of 83 critical factors were identified in the literature from 34 papers. The critical factors are classified into three groups. The first group is related to firm-level critical factors (FLCF), the second to supply chain-level critical factors (SCLCF) and the third to external critical factors (ECF). All three groups of factors play a major role in the success or failure of the implementation of SSCM [1].

##### Firm-Level Critical Factors (FLCF)

Sustainable supply chain management scholars have asserted that firms should consider multiple factors that will enable or hinder the successful implementation of SSCM practices [1]. Several critical factors from various industries and countries have been identified in the literature [1]. Top management commitment and support is considered the most common FLCF [35,38,40]. In ref. [49], the authors have highlighted that top management is responsible for directing sustainability efforts; [50] also found that senior corporate management’s attitude can foster plant-level sustainability management. Indeed, the implementation of SSCM is an internal decision that has to be supported at the firm level [43]. From a supply chain-level perspective, ref. [39] have found that top management is a factor that drives purchasing and supply management sustainability initiatives. On the other hand, low or lack of top management commitment is considered by many researchers as a barrier for the successful implementation of SSCM [1,46]. In the food industry, ref. [34] have found that the most common critical factors for SSCM adoption are the operational cost reduction and market drivers, such as customer requirements, retailer pressure and brand image and corporate reputation. Meeting customer demands, expectations and requirements is one of the most cited critical factors for the implementation of SSCM [35,39,47]. It is widely accepted that customers are the stakeholder group that influences most a company’s performance by buying or rejecting a specific product [51]. For example, there are customers that desire to have environmentally friendly products and services and they are willing to pay more for their demand. If companies fail to meet this specific requirement, they may face customer boycotts [43]. In ref. [39], the authors identified knowledge and expertise regarding sustainability as a driving force for developing an organisation’s SSCM strategy, while ref. [47] highlighted knowledge as a critical intangible asset for SSCM implementation. Indeed, competences, knowledge and expertise are crucial factors for the successful or unsuccessful implementation of SSCM [39,41,43,47]. The recent study of ref. [18] has shown that companies that invest in human capital with professional expertise and capabilities on sustainability issues can enable the implementation of SSCM practices. In the same line, ref. [47] mentions that the lack of knowledge about sustainability issues hinders the development of SSCM. Training and education are other key firm-level critical factors that are closely related to sustainability performance [18]. Training and development about sustainability allow for sustainability improvements in job performance and helps companies minimise errors and waste [18]. A lack of training and education, on the other hand, hinders successful SSCM implementation [1]. In ref. [24], the authors found that despite the fact that social sustainability practices, including participation and training of employees, indirectly impact firm performance, they are positively related. More specifically, social sustainability practices are considered quality-enablers in the food sector [24]. Reputation critical factors are related to brand name and reputation, or minimization of the risk of negative publicity [47]. The authors of ref. [47] highlight that corporate reputation and image are positively related to eco-brand developments. Being proactive regarding sustainability issues can bring a good reputation and image and offer easier market access and develop a good network of suppliers and partners [52]. In ref. [53] (p. 325), the authors further explain that “organizations build a reputation of ‘good citizen’ by promoting environmental and social sustainability in their supply chain. This reputation improves legitimacy and access to key resources”. Firm-level critical factors related to financial issues include cost savings from operational and material efficiencies [47] and the increased resource utilization [39]. On the opposite side, companies that desire to adopt SSCM practices often struggle to overcome the high costs related to the upstream supply chain greening [47] or the development of supply chain infrastructure, systems and processes [19].

##### Supply Chain-Level Critical Factors (SCLCF)

Supply chain-level CFs are closely linked to firm-level CFs. The literature posits that firm level and supply chain-level alignment strongly affect their successful integration [54]. Information sharing has been identified as one of the most important enablers to adopt SSCM practices [38,40,41]. In ref. [18], the authors suggest that information sharing enables the development of new ideas regarding sustainability and enhances collaboration throughout the supply chain. In the food industry, information sharing among supply chain members is described as a novel form for traceability and it is linked to improved supply chain performance [25]. Ref. [34] mentions that product traceability is strongly related to social sustainability and ensures food safety. The limited or lack of information and transparency on sustainability related issues, on the other hand, has a negative impact on SSCM implementation [41,42]. Trustful relationships and commitment among supply chain partners is mentioned as a key factor for implementing SSCM in the food industry. This is due to the criticality of ingredient quality in the food production [41]. According to ref. [34], who investigated sustainability in the Italian meat supply chain, building trust amongst supply chain firms is a core component for implementing exceptional supply chain practices, such as supplier collaboration, for sustainability. On the contrary, ref. [1] highlights that poor supplier commitment is one of the most common inhibiting factors. In ref. [46], the authors found that the lack of trust and commitment between supply chain members is an important obstacle, especially when customers audit suppliers. Agreeing on a common SSCM strategy is another important supply chain critical factor. The authors of ref. [40] found that it is more likely for companies that signal sustainability initiatives to their supply chain partners and stakeholders to develop a common SSCM strategy with them. Developing a common SSCM strategy ensures that all supply chain partners pursue the same strategic goal [40]. Indeed, policy sharing, and the subsequent establishment of common goals, was found to be a key factor for the implementation of SSCM practices such as environmental collaboration [55]. Ref. [11] found that pro-activity is a key factor when pursuing an SSCM strategy in the food industry (e.g., organic food or fair trade) since new processes and technologies need to be established. The lack of agreement on an SSCM strategy hinders the adoption of SSCM. Another factor that significantly affects the adoption of SSCM practices is geographical distance. The findings of ref. [56] show that when geographical distance between suppliers increases, a negative impact is observed on data gathering, assessment and collaboration. More specifically, ref. [41] found that when visiting distant farms or manufacturing plants is required, significant travel effort and resources are needed and as a result it is more difficult to check the partners’ operations and processes. On the contrary, shorter supply chains often lead to the successful implementation of sustainability practices [57].

##### External Critical Factors

External CFs originate from a variety of stakeholders, such as government, customers, suppliers, media, non-governmental organizations (NGOs), etc. Two of the most common external critical factors for SSCM are the existence of regulatory frameworks [38,39,47] and the awareness of and compliance to government policy and legislations [18,35,39,58,59]. Pressure from governments in the form of legislation, such as energy and waste directives, international regulations such as the UN Declaration of Human rights and International Labour Organization conventions, or the EU’s Sustainable Consumption, Production and Sustainable Industrial Policy Action Plan are critical factors for the implementation of SSCM in the food industry [47,52]. Furthermore, pressure from investors [35,47] and interaction with NGOs and other external stakeholders [42] may exert pressure on companies to implement SSCM. Pressures from investors, such as increased investor appeal on sustainability criteria, are considered a driving force to initiate and maintain SSCM [35,47]. Food scares regarding pesticide residues, unhealthy ingredients, chemical residues, etc., result in cautious measures [47]. Other studies have identified competitor’s pressure as a market factor that may lead to the development of SSCM practices. Refs. [35,39,40,47] posit that the adoption of SSCM practices by competitors motivates companies to develop SSCM.

Additional SSCM critical factors are identified in the literature but are not included here, since the concentration in this paper is on those factors that are relevant for sustainable supply chain management in the food industry. A comprehensive list would have to include critical factors such as innovativeness, technology and equipment [18]; employee involvement and traditional accounting methods [35]; additional human resources [42]; personnel commitment [41]; Industry 4.0 solutions [60,61,62], including the Internet of Things (IoT), sustainability data and information [42]; and the supply chain cultural and language differences [41]; among others.

#### 3.1.2. Practices

In ref. [63] (p. 620), supply chain management practice is defined as “a set of activities undertaken in an organization to promote effective management of its supply chain”. In combination with the definition of SSCM that has been provided in the introduction, SSCM practice is defined as a set of sustainability (i.e., economic, environmental and social) activities undertaken in an organization in cooperation with each stakeholders, to promote effective sustainability management of its supply chain. SSCM practices have their origins in green supply chain management (GSCM). Ref. [8] have examined the relationships between GSCM practices and organizational performance in the Chinese manufacturing and processing sectors. In their study they categorized GSCM practices into four groups: (1) Internal environmental management; (2) External GSCM practices; (3) Investment recovery; and (4) Eco-design. Their results have shown that GSCM practices tend to have a positive relationship with environmental and economic outcomes. The same authors three years later used internal environmental management, green purchasing, eco-design, cooperation with customers and investment recovery to represent GSCM practices in their empirical study [64]. Ref. [65] investigated the impact of GSCM practices on organizational performance in the electrical and electronic sector. Their results indicate that green procurement and green manufacturing practices have a positive influence on environmental and financial performance. The authors in ref. [66] identified 47 different logistics social responsibility (LSR) practices and developed a taxonomy of five categories including socially responsible purchasing, sustainable transportation, reverse logistics, sustainable packaging and sustainable warehousing. The authors in ref. [67] have empirically investigated the influence of environmental collaboration practices in the supply chain on environmental and manufacturing performance. In ref. [25], five bundles of SSCM practices were identified through case studies of ten exemplar firms: (1) commonalities, cognitions and orientations; (2) ensuring supplier continuity; (3) re-conceptualize the chain; (4) supply chain management practices including sourcing management, operations and investments in human capital; and (5) measurement. In their list of SSCM practices in the food industry, ref. [24] included both social and environmental issues. More specifically, they have identified four types of SSCM practices, namely, land management, recycling, facility conservation and social practices, and tested their relationships to environmental, quality and cost performance. Focusing on a more social perspective of supply chains, ref. [56] developed a construct of supplier socially responsible practices, including human rights, labour practices, codes of conduct and social audits. In ref. [6], the authors suggest that a positive effect on supply chain sustainability performance could be achieved when firms adopt environmental purchasing and sustainable packaging practices.

The concept of SSCM includes material, information and capital flows; cooperation across the supply chain; economic, environmental and social performance; and customer and stakeholder requirements [2]. The extant body of literature portrays a variety of different SSCM practices, but all have one central objective, namely, the improvement of supply chain sustainability performance. A total of 96 SSCM practices were identified in the literature from 21 papers. In order to conceptualize and develop a sound construct based on the literature and on [11], five practices that cover the aspects of SSCM emerged: (1) strategic orientation; (2) supply chain continuity; (3) collaboration; (4) risk management; and (5) pro-activity. This set of practices emphasizes enhancing the relationships among supply chain partners, the flow of goods and information, and the sustainability aspects.

Despite the major aspects of SSCM that the above practices cover, it should be highlighted that the set of practices that will be described below is not considered complete. Several other practices that have been discussed previously are investigated in the extant literature. In this paper, the SSCM practices as proposed by ref. [11] are used for two reasons: (1) these practices are applied to food supply chains; and (2) the aim of this paper is to further enhance the empirical content of these practices.

##### Strategic Orientation

Strategic orientation refers to the commitment of organizations to SCM, as well as to their dedication to the Triple Bottom Line (TBL) concept [11]. In ref. [25], the authors proposed that, in order to create a sustainable supply chain, a management orientation towards sustainability is required. The balance of environmental, social and economic issues, i.e., the Triple Bottom Line (TBL), plays a crucial role for companies that want to implement a sustainability strategy [68,69,70], and support their decision making [11]. In ref. [21], SSCM practices in the automotive sector were investigated and found that supply chain orientation and the TBL approach are the most important practices for supply chain sustainability. Furthermore, ref. [20] conducted a survey to investigate the impact of SSCM practices from manufacturing companies in various sectors on dynamic capabilities and enterprise performance. Their results showed a positive relationship between supply chain strategic orientation and sustainability performance. In the food industry, ref. [11] found that TBL orientation, which is driven by the consumer’s demand, the company’s motivation and the stakeholders’ pressure, is addressing the sustainability needs of the food industry.

##### Continuity

Supply chain continuity is related to the design and structure of the supply chain network [11]. Ensuring supplier continuity is identified as one of the top sustainable supply chain management practices for exemplar firms [25]. Continuity has to do with the interaction of supply chain members on a permanent base [11]. The core elements of supply chain continuity are the long-term relationships with supply chain partners, the supply chain partner development and the partner selection. Long-term relationships include trust and commitment among the supply chain members [25], which endeavours information sharing [71] and enhances the collaborative design of products or processes [55]. Supplier development refers to the improvement in supplier environmental and social performance [25]. In traditional supply chain management, the development of suppliers is found to be one of the best practices [72], which is also connected to sustainability through mentoring approaches [73]. In the food industry, for example, the assistance and teaching of new farming methods or the funding of costs related to more sustainable farming practices are included in the development of partners [74]. Partner selection is based on their supply chain competency [75] and their desire to develop sustainable practices [76]. Focusing on activities that enhance transparency, traceability, supplier certification and decommodisation is important for ensuring supplier continuity [25]. As ref. [25] (p. 48) describe, organizations that are pursuing continuity in their supply chains, “are trying to ensure that all members of their chain not only stay in business, but that they do so in a manner that allows them to thrive, reinvest, innovate and grow”. Furthermore, focal firms are positively affected by supply chain continuity due to the fact that the supply chain base is stable and capable [25]. Ref. [20] also found a positive relationship between supply chain continuity and sustainability performance.

##### Collaboration

The importance of collaboration in supply chains has been recognized as a key factor but also as a great challenge for supply chain success [77]. Collaboration goes beyond the traditional modus operandi between organisations. First of all, collaboration as an SSCM practice is not restricted only to new product development but also to the development and enhancement of business processes [11,67]. The literature suggests that efficient and responsive supply chains rely on the creation of close and long-term relationships and partnerships with various members of the supply chain in order to increase the customer value [77,78]. Joint development is a key enabler for long-term partnerships. Reference [11] defines it as the collaborative development of new technologies, processes and products. As ref. [79] point out, specific resources from each supply chain partner are required in order to jointly address sustainability issues. The implementation of collaborative development is based on knowledge sharing in order to enable the development of sustainable products and processes [55]. Moreover, suppliers and customers can jointly plan the decrease of their operations’ impact on the environment or support the information exchange and the logistical and technical integration [67]. Collaboration is also characterized by enhanced communication—a very important practice regarding the management of supply chain partners. The quality of information sharing is critical in order to achieve transparency in the supply chain [80,81]. Transparency regarding the origin and ingredients of food, the production methods, etc., is also important for consumers [82]. Despite the need for collaboration to achieve sustainable supply chain management, significant barriers arise that are mainly due to the complexity of supply chains. For example, ref. [77] found that the structure of the food industry and the nature of products have a negative impact on the intensity of collaboration and restrict it to the more tactical-operational, tactical and logistical level.

##### Risk Management

Supply chain risk management includes the adoption of risk mitigation practices to avoid exposure to risks [2]. The adoption of standards and certifications is identified as the most common risk management practice in the literature [11]. This is due to the fact that standards and certifications such as ISO 9001 and ISO 14001 can be applied to a broad range of sectors and they can also be managed (if companies wish) by external consultants, who enhance the level of credibility [83]. Monitoring of specific suppliers in order to explore their needs and identify their progress on specific goals [84] is another practice identified within the risk management category. As authors in ref. [11] mentioned, individual monitoring of suppliers is particularly important in food supply chains, where traceability is a crucial factor to guarantee sustainable production. Despite this fact, individual monitoring is not frequently addressed in the extant literature [11]. Pressure group management is another key characteristic of risk management, which can affect the company’s reputation or performance [85]. In ref. [2], it is pointed out that stakeholders such as NGOs and government should not only be monitored but actively engaged and managed through the implementation of specific practices that address their pressures. It should be noted that the interests of a company and its stakeholders do not always align, and their pressure is seen from a negative perspective [11].

##### Proactivity

Proactivity refers to the actions taken by a company in order to control and manage a specific situation regarding sustainability before it happens, rather than responding to it after it happens. The literature shows that Life Cycle Assessment (LCA) is the most common tool of the pro-activity practice [11]. LCA is used to measure the environmental impacts of the life cycle of a product or service. While LCA is a commonly discussed topic in the literature, ref. [25] found that exemplar firms are using life cycle analysis at the basic level, and only to address the environmental impacts of the chain and not the social ones. Ref. [11] highlights the necessity of supply chain orientation for LCA. If supply chain orientation is not implemented, the information between the supplier, buyer and focal company will not be shared. As a result, joint contributions should be made by all members of the supply chain [11]. Stakeholder management is found to be one of the most frequent practices in the literature [11]. When companies decide to adopt proactive practices, the management of stakeholder requirements is acting as an important factor for performance, products and processes improvement [2,11]. Innovation is another key factor of proactivity and it has been investigated in the field of sustainable supply chain management literature [85]. Innovation includes the capability of a company to generate and implement new ideas and develop or apply new technologies. It is a prerequisite for dynamic market environments such as sustainable supply chain management [11]. An example of supply chain innovation is the adoption of new innovative technologies, such as the Internet of Things or Industry 4.0 tools, which make both internal and external processes more efficient and result in improved sustainability performance [61]. Learning from partners and stakeholders is another important dimension of proactivity. The acquisition of new knowledge is the key characteristic of learning. Companies can learn from supply chain partners, local communities, NGOs, government, researchers, etc. The authors of ref. [86] showed that when firms wish to implement a sustainability strategy, they should be pro-active in the first steps of the product’s development and in its whole life cycle. Overall, ref. [25] highlight that proactivity and commitment can only be effective if companies achieve an alignment between business models and environmental and social sustainability aspects. Ref. [11] further explains that in sustainable food supply chains, such as organic or fair trade, which are dynamic in nature and still young industries, proactive measures are necessary, since many new processes and technologies are under development.

#### 3.1.3. Performance

Sustainability performance refers to how well an organisation achieves its environmental, economic and social goals. Most studies in the literature focus on the economic and environmental performance aspects, whereas the social dimension and the integration of the three sustainability dimensions are still lagging behind [2]. However, the review of [4] revealed a rising interest in studies that investigate the social dimension and the combination of all three dimensions; however, more research is needed in the field. The present section proposes sustainability performance as a three-dimensional construct. A more detailed discussion of the environmental, economic and social performance is provided below. In total, 684 SSCM measures were identified from 55 papers, which were grouped in the following three categories.

##### Environmental Performance

A wide variety of research papers has focused on the environmental performance of supply chains. As ref. [2] argues, this can be explained due to the fact that environmental issues have been on the research agenda for many years. This could be further supported by the fact that, in many countries, organizations are obliged to meet specific thresholds on their environmental impacts; e.g., toxi-chemical releases [87]. The most frequently used measure is related to either the reduction or avoidance of hazardous/harmful/toxic materials. The second most cited measure is water consumption, followed by energy consumption, recycled materials, Life Cycle Analysis (LCA) and environmental penalties. Energy efficiency, air emissions and greenhouse gas emissions are also some of the most cited measures in the literature.

A variety of other measures that appear less in the literature have addressed themes such as waste [79,87,88], environmental management systems, eco-design [89,90], biodiversity [87,91], etc.

##### Economic Performance

Economic performance is typically the most important factor that all companies are aiming to improve. Since the focus of this research is on supply chain management, the economic dimension is an integral part. In the context of SSCM, the comprehensive literature review in [2] shows that economic issues were addressed in all the studied papers. At this point, it should be mentioned that possible trade-offs between the three sustainability dimensions can occur. Especially for the economic dimension, economic incentives could be hidden behind a variety of environmental and social measures [87]. For example, economic performance measures such as procurement costs might increase when deciding to use environmentally friendly materials [4]. The most frequent measure regarding the economic performance is quality. Measures that focus on quality may refer to the quality of products provided by suppliers [87] or to the quality of the production process [73]. Sales, market share and profit are the second most frequent measures, followed by delivery time and customer satisfaction.

Other measures that appeared less in the literature include responsiveness [89,90,92] number of employees [93,94,95], transportation costs [95,96,97], etc.

##### Social Performance

As mentioned before, previous studies have revealed that little research has focused on the social performance of supply chains [12,98]. The authors of ref. [99] argue that this could be due to the fact that social issues are frequently hard to measure. The literature shows that only a few measures are frequently used confirming the fact that little attention has been given to the social dimension of SSCM. The most frequently used measure is recordable accidents followed by training and education and labour practices.

Other social issues that appeared in the literature include human rights [100,101,102], local communities influence [89,90,103], fair trade [57,100,104], philanthropy [105], etc. A recent study [106] has shed light on modern slavery in supply chains, a new area in the agenda of SSCM that has gained a lot of attention lately.

Table 2 lists the proposed constructs described in Section 3.1.1, Section 3.1.2 and Section 3.1.3, along with their definitions and supporting literature.

**Figure 2:**
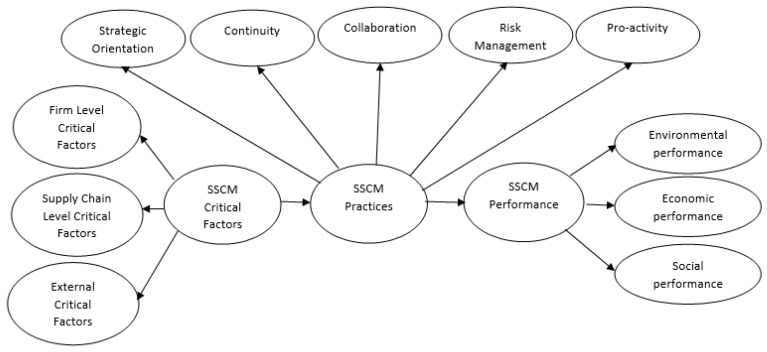
Proposed theoretical framework linking critical factors, practices and performance (based on the literature).

Figure 2 presents the SSCM theoretical framework developed in this study. A detailed description of the identified constructs is provided in the previous sections. Using literature support, this study has linked the developed constructs and proposed the expected relationships among them. The framework proposes that critical factors are influencing the implementation of SSCM practices, which in turn influence SSCM performance. CF is conceptualized as a three-dimensional construct (firm level, supply chain level and external level); SSCM practice is conceptualized as a five-dimensional construct (strategic orientation, continuity, collaboration, risk management and pro-activity); and SSCM performance is conceptualized as a three-dimensional construct (environmental, economic and social).

### 3.2. Case Study Results

The empirical results of the food supply chain case study reflect all the SSCM constructs that have been presented in the theoretical framework. In addition, some new “pioneering” SSCM practices emerged from the data. In Section 3.2.1, the first research question is answered regarding the critical factors for engagement and implementation of SSCM practices. The second and third research questions are answered in Section 3.2.2 and Section 3.2.3, by addressing which SSCM practices are implemented and what measures can be used for SSCM performance measurement in the food industry.

#### 3.2.1. Critical Factors

##### Firm-Level Critical Factors (FLCF)

The commitment and support of top management is reported as predominant firm-level critical factor for SSCM implementation. As highlighted, “sustainability is seen as an integral part for the future of our business. You cannot produce like there is no tomorrow, you produce because you want tomorrow to exist” (CSR Manager, SB). SSCM requires “proactive top management that understands that sustainability is an organizational commitment” ([25], p. 40). Indeed, top management is a critical firm-level factor for the promotion of SSCM and its absence may act as an obstacle for SSCM adoption [1,35].

Customer-driven orientations, in order to meet customer demands and needs, have been confirmed as critical factors of SSCM implementation, by all the interviewees. Previous studies have found that customer demands and requirements drive the development and implementation of SSCM practices [35,39,47]. For example, ref. [19] found that customer expectations are some of the most important driving forces for SSCM implementation. Similarly, ref. [47] have confirmed that customer demand and expectations are market drivers for corporate supply chain responsibility. In other words, adapting to what customers want is necessary for the implementation of SSCM at all supply chain stages.

According to the managers and the companies’ records, expertise and knowledge on environmental and social issues of supply chains is required to implement SSCM practices. Knowledge about how suppliers and other partners work regarding sustainability, is a critical SSCM factor that exemplar firms are adopting to improve their entire supply chains [25].

Employee training and development was also confirmed by both companies as another important firm-level critical factor. All key informants highlighted the continued efforts of their companies to offer a variety of programs in order to improve employee satisfaction and raise the sustainability awareness. Training and development can lead to engagement in SSCM practices, which, as mentioned by the interviewees, is a crucial part of the corporate strategy. It was evident by both the participants and the companies’ records that training and development programmes improve job performance and reduces errors and waste, which was also confirmed by the study of [18].

Efficiency in operations and material management was mentioned by the participants. Efficient energy management and electricity generated from renewable energy sources were the top mentioned factors of SSCM. Another element of efficiency is technology. Both companies exploit the available technologies to improve and optimize operational processes. This leads to cost savings and resource reduction and thus offers the ability for new investment plans.

##### Supply Chain-Level Critical Factors (SCLCF)

The sampled supply chain is involved in traceability actions with their suppliers. Previous literature suggests that traceability is a new form of information sharing [25]. There is a requirement for information sharing on the living conditions of the animals, on the production of products, on the materials used, the locality information, information related to product labelling, etc. As reported, clear information about the products and their ingredients are provided on the front and back of the packages.

According to the interview data, the key to successful solutions to the daily problems is trust. Trusted partnerships and long-term cooperation build relationships of trust and confidence with suppliers. In this way, both companies achieve their goals, while at the same time “pushing” their suppliers to develop and improve as individuals. The same logic applies to the customers as well. Several systems are applied in the sampled companies, such as, compliance management system as well as anti-corruption and antifraud systems. In general, both companies are trying to create a climate of mutual trust among their stakeholders (employees, customers, suppliers, local communities, etc.).

Both companies have managed to establish a common supply chain strategy with their supply chain partners. The improvement in environmental and social standards across the supply chain is in the core element of the SSCM strategy. The organisations implement a sustainability strategy in their partnerships that includes goal-oriented actions. As reported, the suppliers are a crucial part of the supply chain, and through continuous dialogue with them, the added value of the products and services reducing one’s environmental footprint and effects on society is enhanced. Geographical distance was not mentioned by the participants. However, both companies use local sourcing in more than 80% of their operations. This creates additional added value in the local economy, with the indirect creation of jobs.

##### External Critical Factors

The interview data revealed that legislation requirements very often force companies to transform their business by applying sustainable practices; e.g., water saving. This is further identified in the secondary data, where strong focus is given on information regarding the legal penalties or fines for non-compliance with environmental and social regulations. Information is also provided regarding the compliance to European and national legislation on consumer products and the non-promotion and communication to minors (aged under 18 years old). The literature suggests that regulations and legislations can act as strong driving forces for the implementation of SSCM practices [39,43]. Examples, such as the British Petroleum (BP) oil spill, have shown that there is as huge negative impact on the supply chain economic performance, estimated at around $90 billion, including civil and criminal penalties [107].

Furthermore, the trends of stakeholders undoubtedly constitute an important pressure as they can also change a company’s strategy. For example, an interviewee mentioned that when there was an intensive debate about obesity, the company realized that it could not ignore it and decided to develop new products for consumers who do not want to get extra calories. In this way, the consumer had the choice of choosing the suitable product regarding his/her wishes. The sampled companies engage stakeholders in active dialogues throughout the year, to determine and redesign their sustainability strategy and actions and understand how to meet their needs and expectations. Stakeholder management is critical for maintaining a healthy and sustainable business. As declared with the CSR reports of the two companies and corroborated by the interview data, producing a positive value for stakeholders and creating the conditions for a healthy competitive environment enhance sustainable development.

The risk of changing product quality after production is mentioned as a key external critical factor for implementing SSCM practices. As reported, a company makes significant investments in order to offer the customer the right product, in the right package, at the right point of sales and at the right price, with its primary concern being safety. Another interviewee highlighted that the company is developing and implementing systems, standards and practices to ensure food quality and safety and avoid actual and reputational risks such as child labour.

#### 3.2.2. Practices

The data analysis suggests the development of two main groups of practices: the traditional SSCM practices and the pioneering SSCM practices. The first group includes the SSCM practices as identified in the literature, while the second group encompasses SSCM practices that are adopted by leaders. The term “pioneering” is used only to describe these practices in the Greek food industry context. In the following sections, a description of both groups of practices is provided.

##### Traditional SSCM practices

Collaboration

Collaborations with supply chain members such as suppliers and customers as well as with a range of stakeholders such as NGOs and other entities are identified as key practices that help both companies and their supply chains to achieve sustainability goals. Long-term collaborations and contact with suppliers and stakeholders create relationships of trust and confidence. Development and improvement of suppliers as individuals is another characteristic of collaboration that emerged from the data. Joint development and training of suppliers is found to add value in the supply chain management performance. The data revealed that the companies are already deploying traceability practices for specific products. In parallel, they both are in the process of digital transformation, which will help them to increase supply chain traceability, transparency, quality, speed and efficiency.

Continuity

Practices regarding suppliers’ and external partners’ selection are reported in the continuity category. According to an interviewee from SB, “There are guiding principles for all suppliers which include a wide range of requirements such as the confirmation that children are not working at a supplier’s company”. SB is implementing a “continuous development” approach, which deploys corrective actions to ensure that all suppliers comply with the company’s environmental, social and labour policy. Furthermore, the data suggest that partnering with reliable suppliers, especially in quality and safety issues, is necessary for a continuous relationship.

Strategic orientation

As reported, both companies are engaged in strategic supply chain management, which promotes the balance among environmental, economic and social issues. The data reveal that an SSCM strategy was already in place and three common characteristics were identified. First, a continuous business model alignment with economic, environmental and social issues is in place. For example, SB has re-designed a series of their products towards reducing plastic in packaging and this resulted in environmental and economic benefits, while at the same time allowed the company to apply similar techniques to other products. This is consistent with previous studies that found that alignment of environmental, social and economic goals is needed for managerial orientation towards sustainability [25]. The second and third component is that both companies treat suppliers as key strategic partners and focus on strategic sustainability issues related to the local communities.

Risk management

The implementation of management systems is used as a risk management tool for both companies. Food quality management systems (e.g., ISO 22000), environmental management systems (e.g., 14001) and health and safety (OHASAS 18001) are identified as key risk analysis tools. Furthermore, a strict supplier selection criteria system is supporting the risk management practices along with supplier monitoring through tactical inspections. Apart from the risk mitigation outcomes, tactical inspections are a pre-requisite for the successful interaction and long-term relationships among the supply chain members.

Proactivity

In this group of practices, the key component is to go beyond compliance with current legislation requirements by engaging in more advanced sustainable practices. Product innovation (e.g., products with reduced calories) and process innovation especially in the logistics domain are identified as key for SSCM. Supplier codes of conduct, including environmental, health and safety, labour and social issues, as well as partners’ coaching to adopt and implement SSCM practices are also included in proactive practices. Finally, energy- and water-saving practices and efficient fleet management are implemented to reduce the negative outcomes. Another set of practices that is related to proactivity, as stated by the CSR Manager of the SB and the Logistics Project Manager of the SM, is employee welfare, human rights practices, and the supporting actions for young people and local communities.

##### Pioneering SSCM Practices

Conversation

Sustainability is part of the daily conversation in the two companies. Discussions of noneconomic issues is shared across all departments. As the CSR Manager of the SB company mentioned, “the basic principle in our company is social and environmental responsibility in our daily transactions”. Daily conversations about sustainability issues are part of all decision-making processes in a way that all employees consider social and/or environmental impacts of their decisions. As ref. [25] (p. 51) proposed, “management orientation is evidenced by sustainability being part of the day-to-day conversation”.

Local sourcing

Local sourcing was evidenced by a focus on sourcing from Greek suppliers in more than 80%. Clear sustainability benefits of local sourcing include minimization of transport, increase of freshness and contributions to environmental and social improvements.

Investing in Human Resources

Investing in human resources is considered a key SSCM practice. As in previous studies on sustainability leaders [25], the internal focus in this sample is on employee investments. Both companies provided information regarding their programmes for employee training, skills development and benefits. They both recognised positive outcomes regarding the employees’ personal development and well-being and their commitment to the organisations’ goals. As an interviewee mentioned, “investing in employee training and development not only serves as a motivation, but it also enables the organization to create a highly skilled workforce”.

#### 3.2.3. Performance

By analysing the companies’ records, it became evident that sustainability performance was measured through specific indicators and standards. More specifically, both companies follow the GRI and UN Global Compact principles. This is evidenced by the sustainability reports, which reveal that the companies are adapting to international sustainability reporting standards. This should be no surprise, since both companies are sustainability leaders.

##### Economic Performance

Both companies have mentioned that SSCM is related to a direct increase in costs. Many of the aforementioned practices, apart from the financial resources, include investments in human, and time resources. For example, practices regarding suppliers’ and external partners’ selection, such as the suppliers guiding principles of the SB, which require the confirmation that children are not working at the supplier’s company, as well as the tactical supplier inspections, increase costs. However, as the CSR Manager of SB mentioned, “sometimes you pay more to have the best suppliers and this contributes to added value for costumers, which increases customer loyalty”. Supporting local suppliers to adopt SSCM practices (employee protection and security, human rights, etc.) also contributes to the local economy through indirect job creation.

On the contrary, energy-saving practices are found to have a positive financial impact by means of cost reduction, which increases the profit rates. This is due to the fact that energy-efficiency investments are producing results from the first day of implementation. For instance, both companies have invested huge amounts in LED lighting, which is considered a highly energy-efficient technology.

Quality improvement is another important economic factor that both companies are engaged in. For instance, SM has mentioned that compliance with quality standards and reduction of defective products are key quality measures.

Not surprisingly, sales and market share, is also found to be a key economic measure. Other measures discussed under the economic dimension are the annual R&D investments, productivity, delivery time and flexibility.

##### Environmental Performance

As expected from both companies, as sustainability leaders, they have environmental performance systems in place that manage not only the environmental “basic” indicators (hazardous/harmful/toxic materials, energy, water, CO_2_ emissions, compliance to standards, environmental accidents and use of recycled materials) but the advanced ones as well, such as the re-design of products towards a reduction in plastic and the reuse of it through circular processes. A key characteristic of both companies is that most of the indicators are measured at the organizational level. For example, energy use is measured in both companies’ facilities but not in their suppliers’ operations. It is also reported that the energy consumed comes from renewable energy sources at a level of 100% in SB’s facilities and 97% in SM’s facilities. Managers from SM have reported that the company is planning to measure the indirect emissions of its supply chain. As [9] propose, a useful tool to measure the impact of a supply chain as a whole is life-cycle analysis (LCA).

A variety of other measures have addressed themes such as waste recovery [20], waste [79,87,88], environmental management systems, eco-design [7,89,90] biodiversity [87,91], etc.

##### Social Performance

In the social sustainability dimension, the data suggested indicators such as product safety, employee accident rates, employee training rates, health and safety issues, employment contribution, employee benefits, loyalty and turnover rate, corporate image, human rights screening (suppliers and contractors) and community support. Several projects both internal and external are implemented in both companies. For example, an excellent working environment that is fair, safe and enjoyable with prospects for development (such as job rotation, promotions, new roles, etc.) is a key performance measure for SB. From an external point of view, supplier social assessment is performed from SB regarding the suppliers’ human right policies and broader social issues. Furthermore, SM reported that local community support in the form of volunteering or charity actions is another key performance indicator.

Table 3 presents the SSCM aspects as identified in the case study. 

### 3.3. Discussion

**Figure 3:**
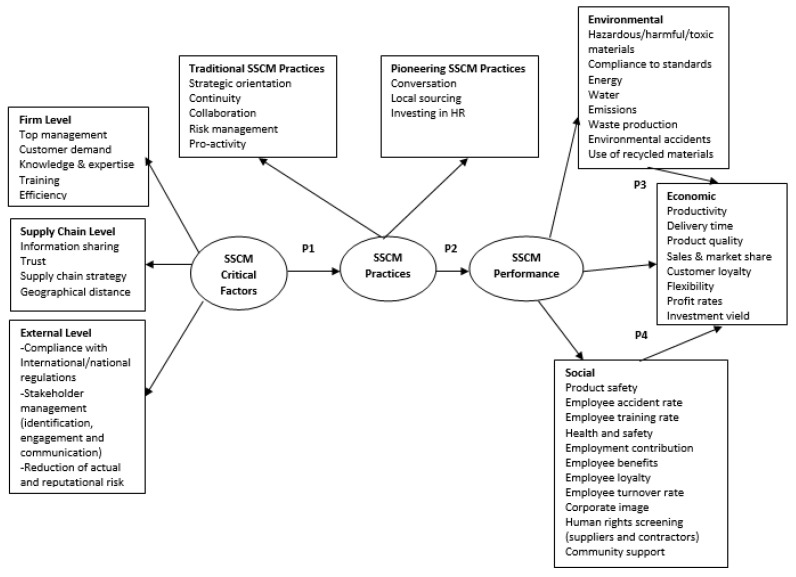
Conceptual model of sustainable supply chain management in the food industry.

The results of this study offer empirical evidence regarding the identified constructs and their interrelationships. More specifically, the data analysis suggests a model of SSCM in the food industry, providing a first step toward defining three constructs (critical factors, practices and performance) that can create sustainability in the food industry. The proposed model is depicted in Figure 3.

The model is developed based on the extant literature and the case study data. Figure 3 presents specific relationships between the constructs, which contribute to a better understanding of SSCM in the food industry. In the following paragraphs, the relationships of the proposed constructs are conceptualized in propositions that need to be tested in future research.

The ability of a company to identify and understand the factors that enable and inhibit the creation of sustainability across supply chain is critical for SSCM. A variety of SSCM critical factors is identified and categorized at the firm level, the supply chain level and the external level. These factors are linked to the implementation of SSCM practices. In line with prior literature, the commitment of top management or the knowledge and expertise regarding sustainability are identified as important firm-level critical factors for SSCM. For example, ref. [1,35] suggest that the lack of top management commitment and support hinder the development of SSCM. SSCM requires “proactive top management that understands that sustainability is an organizational commitment” [25] (p. 40).

At the supply chain level there is evidence that information sharing and trust between partners are two of the key critical factors for implementing SSCM. The literature posits that that information sharing enables the development of new ideas regarding sustainability and enhances collaboration throughout the supply chain [18]. On the opposite side, the lack of information sharing is found to have a negative impact on SSCM implementation [41,42].

Regarding the external environment, three key factors have been confirmed by the dataset: compliance with international and national regulations, stakeholder management and reduction in actual and reputational risk. The identification, engagement and communication with customers, local community and NGOs were reported as critical factors for the successful implementation of SSCM practices. This is consistent with prior literature which confirmed that stakeholders are driving forces for the integration of SSCM practices [19]. Especially in the food retail industry NGO pressure is critical for the adoption of SSCM [53].

Based on the above, the first set of propositions is developed below.

**Proposition** **1.** *SSCM critical factors are directly related to the implementation of SSCM practices.*

**Proposition** **1a.** *Firm-level critical factors are directly related to the implementation of SSCM practices.*

**Proposition** **1b.** *Supply chain-level critical factors are directly related to the implementation of SSCM practices.*

**Proposition** **1c.** *External critical factors are directly related to the implementation of SSCM practices.*

Considering the adopted SSCM practices, the findings suggest two main groups, namely, the traditional SSCM practices and the pioneering SSCM practices. Traditional SSCM practices include the five categories proposed in the literature. This is not a surprise, since the sample of this study is comprised by leaders in sustainability. In this case study, the SSCM practices as proposed by [11] are used as a key starting point and as a guiding tool for developing a model of SSCM in the food industry. What is interesting in this case study, is the possible trade-offs between the SSCM practices. For example, the focus on supplier continuity requires long-term relationships which is a key element of collaboration. This is also consistent with prior literature which suggests that supply base continuity long-term relationships are critical for the successful implementation of SSCM [108]. Continuity was also evidenced by a focus on supplier risk management. Both companies have in place a supplier selection criteria system, which is also related to the supplier codes of conduct that comprise environmental, health and safety, labour and social issues. Regarding the three identified pioneering SSCM practices (conversation, local sourcing and HR investments), it should be noted that they could have been encompassed in the traditional SSCM practices. However, it was decided to be separately presented since both companies engage in these practices in significant amounts. Furthermore, the purpose was to show what sustainability leaders in the food industry are doing regarding SSCM. In no way do these three practices constitute something new or unique.

The findings underline that SSCM performance is linked to SSCM practices. Despite the fact that all participants agreed on a direct increased cost of implementing SSCM, their general perspective was that SSCM practices have the ability to enhance environmental and social performance. This is also supported by [6], who found that environmentally friendly purchasing and sustainable packaging have a positive effect on sustainable performance. Another example based on the results is food safety, which is linked to improved sustainability and can be achieved through traceability practices. Evidence of similar results is also provided by [34], who found that traceability practices in the meat supply chain are closely associated with social sustainability and food safety. It can also be argued that traceability is the end-result of sharing information, which is related to enhanced supply chain performance [25]. Based on the above observations, the following propositions are developed.

**Proposition** **2.** *SSCM practices are positively associated with sustainability performance.*

**Proposition** **2a.** *Strategic orientation is positively associated with sustainability performance*.

**Proposition** **2b.** *Continuity is positively associated with sustainability performance.*

**Proposition** **2c.** *Collaboration is positively associated with sustainability performance.*

**Proposition** **2d.** *Risk management is positively associated with sustainability performance*.

**Proposition** **2e.** *Pro-activity is positively associated with sustainability performance.*

**Proposition** **2f.** *Conversation, is positively associated with sustainability performance.*

**Proposition** **2g.** *Local sourcing, is positively associated with sustainability performance.*

**Proposition** **2h.** *Investing in HR is positively associated with sustainability performance.*

Another interesting finding is the interrelationships between the three dimensions of sustainability performance. The data suggest that environmental performance improvements, such as energy efficiency practices, have visible cost reductions in the short term. This contradicts the results of [24], who found that in the food industry environmental performance is not affecting costs directly. Continuing with a study in the Italian meat supply chain, ref. [34] found that SSCM practices, such as cleaner technologies, offer a competitive advantage, since they contribute to improved economic and environmental or social performance. Ref. [109] also found a positive correlation between corporate social performance and corporate financial performance. Based on the above arguments, the following propositions are developed.

**Proposition** **3.** *Environmental performance is positively associated to economic performance.*

**Proposition** **4.** *Social performance is positively associated to economic performance.*

## 4. Conclusions

### 4.1. Theoretical Contributions

This research has examined the SSCM critical factors, practices and performance through a literature review and a case study comprised of sustainability leaders in the food industry. The study has identified the SSCM critical factors and practices that sustainability leaders implement and what measures are used in sustainability performance in the food industry. In line with ref. [32], who highlights the deductive nature of case studies, this research investigated the applicability and validity of the three SSCM constructs as identified in the literature review, in a specific Greek food supply chain. The case study implies direct and indirect links among the three key constructs, namely, SSCM critical factors, SSCM practices and sustainability performance. Furthermore, in line with the developed propositions, the three constructs are conceptualised within a model that needs to be quantitatively tested.

It can be argued that it is not a surprise that the two sustainability leaders are more committed to SSCM. Both have identified common factors that are critical for developing SSCM practices. This study has also identified a new set of pioneering SSCM practices in the Greek food industry. Daily conversations, local sourcing and investing in HR are common practices for SSCM leaders in the Greek food supply chain, however industry specific.

The developed SSCM conceptual model can be exploited by researchers that wish to investigate the proposed constructs individually or together, both at the firm level and the supply chain level, and either through quantitative (surveys) or qualitative research methods (replicate the case study in other geographical locations or other industries). Researchers may also take advantage of the developed model and use it as an evaluation framework or as an SSCM roadmap for the design of future research projects.

### 4.2. Managerial Implications

Apart from the theoretical contributions, this study provides some managerial implications regarding the deployment of the proposed model. While the identified constructs in this research are not new and can be characterized as SSCM traditional, they have been studied in a food supply chain considering all the three sustainability dimensions. The developed model can be used by companies in the food industry that want to promote or determine the best way to develop SSCM and improve their sustainability performance. The results can be utilized by food industry professionals and assist them in the development of SSCM by identifying the critical factors of SSCM implementation, the practices adopted, and the sustainability performance measures.

### 4.3. Limitations and Future Research Directions

This study, as in any other research, suffers from limitations that will be presented along with future research propositions. First, the sample is small, industry and location specific, and the results cannot be transferred or used to generalize the overall food industry. Future studies may conduct research in other industries or world regions, using larger samples, in order to achieve generalization of the results. Second, this study focused on food sustainability leaders. It is likely that in more typical organisations—not sustainability leaders—different SSCM factors, practices and performance measures will be identified. Third, the traditional and pioneering practices should be investigated in other industries to check their applicability as well as the possible trade-offs. Finally, in this study, specific interrelationships among the constructs are addressed. However, the small sample does not allow for deeper investigations. Future research should examine the importance of each of the constructs and the strength of their inter-relationships.

## Figures and Tables

**Table 1:** Sample characteristics.

Company	Description	Size/Ownership
Soft drinks and beverages (SB)	Multinational producer and distributor of soft drinks and beverages	Large/Private
Super Market (SM)	Multinational distribution centre and retailer	Large/Private

**Table 2:** Proposed SSCM constructs, along with their definitions and supporting literature.

Constructs	Definitions	Reference
SSCM Critical Factors		
Firm-Level Critical Factors	Firm-level critical factors refer to internal factors that firms should take into consideration for the successful implementation of SSCM practices. Top management commitment, customer demand, knowledge and expertise, training and efficiency are some of the most common firm-level critical factors for SSCM.	[1,18,19,35,38,39,40,41,43,47,50,53]
Supply Chain-Level Critical Factors	Supply chain-level critical factors are closely linked to firm-level critical factors and refer to the supply chain’s motivational activities that promote the implementation of SSCM practices. Some of the most common supply chain-level critical factors identified in the literature are information sharing, trust, supply chain strategy and geographical distance.	[1,35,38,40,41,56,57]
External Critical Factors	External factors refer to the external considerations that firms do not control but, should take into account for the successful implementation of SSCM practices. Government policy, international/national regulations, stakeholders, competitors, investors and food incidents are identified as some of the most common in the SSCM literature.	[17,35,38,39,40,41,42,47,48,58,59]
SSCM practices		
Collaboration	Supply chain collaboration is dealing with the design and the government of supply chain activities as well as the establishment and maintenance of long-term supply chain relationships. Collaboration allows the joint development, the technical and logistical integration, the enhanced communication and the knowledge and information sharing among supply chain partners.	[11,67,77]
Continuity	Supply chain continuity refers to the design and structure of the supply chain network in order to achieve successful interaction of supply chain members on a permanent base. Key characteristics include the long-term relationships with supply chain partners, the partner development and selection.	[11,20,25]
Strategic orientation	Strategic orientation refers to the commitment of organizations to supply chain management, as well as to their dedication to the Triple Bottom Line (TBL) concept, which promotes the balance of environmental, social and economic issues.	[11,25,68,69,70]
Risk management	Supply chain risk management includes the adoption of risk mitigation practices to avoid exposure to risks. The adoption of standards and certifications, the monitoring of supply chain partners and the engagement of stakeholders are some of the key practices.	[2,11]
Pro-activity	Proactivity refers to the actions taken by a company in order to control and manage a specific situation regarding sustainability before it happens, rather than responding to it after it happens.	[11,25]
SSCM Performance		
Economic	Economic performance refers to how well an organisation achieves its economic goals. Productivity, delivery time, product quality, sales & market share, customer loyalty, flexibility, profit rates and investment yield are some of the most frequently used indicators to measure economic performance.	[6,73,79,87,88,89,90,91,92,94,95,97,103]
Environmental	Environmental performance refers to how well an organisation achieves its environmental goals. Hazardous/harmful/toxic materials, compliance to standards, energy, water, emissions, waste production, environmental accidents and use of recycled materials, are identified as the most common environmental performance indicators.	[6,8,79,87,88,89,97,103]
Social	Social performance refers to how well an organisation achieves its social goals. Product safety, accident rate, training rate, health and safety, employment contribution, benefits, loyalty, turnover rate, corporate image, human rights screening (suppliers and contractors) and community support have been identified in the literature as some of the most common social performance measures.	[20,93,94,97,99,105]

**Table 3:** Aspects of SSCM as identified in the case study.

Constructs	SSCM Aspects as Identified in the Case Study
Critical Factors	
Firm Level	Top management beliefs and behaviours related to sustainability is a key starting point in order to create sustainable supply chains Building a customer-driven sustainability orientation is a key factor for developing SSCM Knowledge and expertise on sustainability issues is at the core of SSCM. This is the way to overcome sustainability challenges. Employee training and development programs including social and environmental issues should be embedded across the organization. Efficiency in operations and resources through the use of state-of-the-art technologies is a critical factor for SSCM.
Supply Chain Level	Traceability and transparency are considered key sustainability factors for providing crucial information about the product’s safety, environmental footprint and animal welfare. Information sharing through traceability helps companies identify supply chain risks. Creating and maintaining trust with customers, suppliers and local communities, through the development of systems and standards is critical for building a sustainable supply chain. A common goal-oriented sustainable supply chain strategy, through the continuous dialogue with supply chain partners, is enhancing the added value of products and services. Geographical distance enables the development of SSCM. Both companies source from local suppliers to support the local economy, improve quality and minimize transport
External Level	Compliance with government policy, international and national regulations is a prerequisite for developing SSCM. Stakeholder management includes internal and external customers, suppliers, business associations, NGOs, local and governmental authorities. The companies use several tools of stakeholder engagement such as customer and supplier surveys, focus groups, emails and personal meetings. The SB company has identified itself as a key stakeholder and the analysis is following the same procedure as in other stakeholder groups. Reduction in actual and reputational risk (e.g., child labour) is triggering SSCM.
SSCM practices	
Traditional practices	
Collaboration	Partnership and long-term collaboration and contact builds relationships of trust and confidence with suppliers. Collaborating with supply chain members helps in achieving supply chain sustainability goals while at the same time contributes to the development and improvement of suppliers as individuals. Joint development and training (e.g., on recycling practices) is implemented to foster the supply chain added value. The companies are deploying traceability practices for specific products. They are in the process of digitalizing their procurement/supply chain systems in order to increase supply chain traceability, transparency, quality, speed and efficiency.
Continuity	A “continuous development” approach is adopted by SB which is implementing corrective actions trying to ensure that all suppliers comply with the company’s environmental, social and labour policy. Partnering with reliable suppliers especially in quality and safety issues is necessary.
Strategic orientation	Aligning the business model with sustainability considerations Treating suppliers as key strategic partners Focusing on strategic issues related to the local community
Risk management	The adoption of management systems is used as a key risk management tool to mitigate supply risks Strict supplier selection criteria system Supplier monitoring (tactical inspections)
Pro-activity	Going beyond compliance with current legislation requirements by engaging in more advanced sustainable practices. Supplier codes of conduct including environmental, health and safety, labour, social issues, etc. Coaching potential partners to adopt and implement practices and initiatives aiming at sustainable supply chain development Energy-saving practices Water-saving practices Efficient fleet management
Pioneering practices	
HR investments	Investments in human capital through several programs and actions is part of the companies’ organizational culture
Daily conversation	Discussions of environmental and social issues is shared across all departments in a systematic way. Sustainability is not an occasional issue
Local sourcing	80% of sourcing comes from Greek suppliers
SSCM Performance	
Economic	Increased direct costs Reduced energy costs Productivity Delivery time Product quality Sales and market share Added value for customers Increased customer loyalty Flexibility Profit rates Investment yield
Environmental	Minimization of hazardous/harmful/toxic materials Energy savings Water savings CO_2_ Emissions Waste production Use of recycled materials
Social	Improved product safety Improved employee welfare Employee accident rate Employee training rate Health and safety Employment contribution Employee benefits Employee loyalty Employee turnover rate Corporate image Human rights screening (suppliers and contractors) Community support

## Data Availability

The new data that were created and analyzed in this study are the ones presented in the article. Interview or field notes data sharing is not applicable to this article.

## Appendix A

### Interview Protocol

(1) General information about the company
(2) Critical factors of SSCM
  - What are the factors that push the company to implement SSCM practices?
  - What are the factors that hinder the company to implement SSCM practices?
(3) SSCM practices:
  - What are the SSCM practices implemented in your company?
    Strategic orientation
    Supply chain continuity
    Collaboration
    Risk management
    Pro-activity
(4) Impact on performance:
  - What measures/indicators does your company use to measure SSCM performance?
  - How has the implementation of SSCM practices affected the environmental, social and economic performance of your company?
  - Is there any observed relationship between environmental, social and economic performance (win–win, win–lose)?
